# Balanced selection on purebred and crossbred performance increases gain in crossbreds

**DOI:** 10.1186/s12711-018-0379-9

**Published:** 2018-03-22

**Authors:** Hadi Esfandyari, Peer Berg, Anders Christian Sørensen

**Affiliations:** 1 Department of Molecular Biology and Genetics, Center for Quantitative Genetics and Genomics, Aarhus University, Tjele, Denmark; 2 Norwegian University of Life Sciences, Ås, Norway

## Abstract

**Background:**

Genomic selection can be applied to select purebreds for crossbred performance (CP). The average performance of crossbreds can be considered as the summation of two components, i.e. the breed average (BA) of the parental breeds and heterosis (H) present in crossbreds. Selection of pure breeds for CP based on genomic estimated breeding values for crossbred performance (GEBV-C) or for purebred performance (GEBV-P) may differ in their ability to exploit BA and H and can affect the merit of crossbreds in both the short and long term. Selection based on GEBV-C is beneficial for CP, because H in crossbreds is efficiently exploited, whereas selection on GEBV-P results in more genetic progress in pure breeds, which increases the BA component of CP. To investigate the outcome of selection on GEBV-C and GEBV-P in both the short and long term, a two-way crossbreeding program was simulated to test the following hypotheses: (1) does selection on GEBV-P result in higher long-term CP compared to selection on GEBV-C and (2) does selection on a combination of GEBV-P and GEBV-C lead to more long-term gain in CP than selection on either separately.

**Methods:**

We investigated the performance of crossbreds in a two-way crossbreeding program across 40 generations and considered different criteria to select purebred parents that ranged from selection on purebred performance to selection for CP with different weights on genomic evaluations based on purebred and CP. These criteria were compared under three genetic models to investigate the effects of the amount of dominance variance, absence of over-dominance, and the structure of the reference population on CP, both in the short and long term.

**Results and conclusions:**

Although beneficial in the short to medium term, genomic selection in pure breeds on a criterion that specifically targets CP was inferior to selection for purebred performance in the long term. A selection criterion that maximizes a combination of short- and long-term responses in CP, should improve the components that define crossbred merit (i.e., BA and H) simultaneously.

**Electronic supplementary material:**

The online version of this article (10.1186/s12711-018-0379-9) contains supplementary material, which is available to authorized users.

## Background

Genomic selection can be applied to select purebreds for crossbred performance (CP) [1–3], by estimating effects of single nucleotide polymorphisms (SNPs) based on phenotypes and genotypes from either purebreds or crossbreds, and applying the resulting estimates to selection of purebreds [1]. Several genomic models have been suggested for the prediction of breeding values for CP of individuals in the pure breeds in genomic crossbreeding programs. These models are the standard additive genomic prediction model, models with across‐breed effects of SNP genotypes (ASGM) or with breed‐specific effects of SNP alleles (BSAM) [2], the dominance model [3], and the breed-specific dominance model (BSDM) [4]. Additive and dominance models can be applied to either crossbred or purebred performance, while ASGM, BSAM and BSDM can only be used for training on crossbred performance.

It has been suggested that, to improve CP, selection of purebred animals should be based on genomic estimated breeding values (GEBV) that have been calibrated based on allele frequencies of the opposite breed rather than based on allele frequencies observed within the breed [1, 5]. The logic behind this originates from the fact that in crossbreeding, alleles at quantitative trait loci (QTL) from the sire (dam) breed combine with alleles from the dam (sire) breed. When dominance is present, allele substitution effects and breeding values depend on the allele frequency among the mates [6]. Since dominance is a likely genetic basis of heterosis, selecting purebred animals for CP based on GEBV for CP (GEBV-C) that are calculated using both additive and dominance effects and appropriate allele frequencies, is expected to increase heterosis. Although GEBV-C is beneficial for CP, it is expected to reduce genetic improvement within the pure breeds [7], which is more pronounced if QTL with over-dominance affect the trait. In such a case, CP is maximized if alternate alleles are fixed in the two parental breeds. In fact, with over-dominance, allele substitution effects may have opposite signs in the parental breeds, depending on their allele frequencies. Since GEBV-C are based on the allele frequencies in the gametes that are produced by the opposite breed, the two parental breeds are expected to be fixed for alternate alleles of over-dominant QTL, which maximizes the frequency of favorable heterozygotes in crossbred progeny. However, fixation of alleles that are not favorable for purebred performance will reduce genetic improvement in the pure breeds.

The average performance of crossbreds can be written as the summation of two components i.e. breed average (BA) of pure breeds and amount of heterosis (H) present in crossbreds (CP = BA + H) [6]. Criteria to select pure breeds for CP such as GEBV-C or GEBV for purebred performance (GEBV-P) can differ in their ability to exploit these two components. Selection on GEBV-C is beneficial for CP, because it exploits H in crossbreds efficiently, while selection on GEBV-P can result in more genetic progress in pure breeds, which increases the BA component of CP. In a two-way crossbreeding system, Esfandyari et al. [7] showed that selection of pure breeds on GEBV-C can increase response in CP compared to selection on GEBV-P. In their study, the comparison between the two selection criteria was limited to five generations. However, in practice the goal is to maximize gains in current and future generations. Several studies have shown that maximization of short-term response may result in smaller cumulative responses in the longer term in the context of pure breeding [8–10]. In crossbreeding programs, it is also unclear how GEBV-P and GEBV-C affect the two components of CP (i.e. BA and H) in the long term. In fact, the main reason for the superiority of GEBV-C for CP in the short term is its ability to drive the QTL with over-dominance effects towards fixation of alternate alleles in the two parental breeds [7]. Once those QTL are fixed, subsequent improvement in CP can be obtained only by genetic gain in BA, for which selection on GEBV-C is inferior to selection on GEBV-P. Therefore, to investigate long-term effects of selection for CP, we simulated a two-way crossbreeding program to test the following two hypotheses: (1) does selection of purebred on GEBV-P result in greater CP in the long term compared to selection on GEBV-C, and (2) does a criterion that combines GEBV-P and GEBV-C result in greater long-term gain (CP) than selection on either separately.

## Methods

### Procedure

We analyzed 40 generations of selection in a two-way crossbreeding program to improve performance of crossbred progeny. We compared five genomic selection criteria in the context of three genetic models to investigate the effects of the amount of dominance variance, absence of over-dominance, and structure of the reference population (purebred or crossbred) on short- and long-term response in CP. Simulations were performed using the *xbreed* package and scripts were developed in R [11]. Each scenario was replicated 50 times.

### Selection criteria

Improvement in CP was examined by using five genomic selection criteria that differed in the approach used for the selection of purebred animals as the parents of both crossbreds and of the next generation of purebred animals. The general selection criterion for animal *i* within each parental breed was:

$$SC_{i} = \left( {1 - w} \right) \cdot {\text{GEBV}}_{iP} + w \cdot {\text{GEBV}}_{iC} ,$$

where, ${\text{GEBV}}_{iP}$ is the GEBV of animal *i* for purebred performance, ${\text{GEBV}}_{iC}$ is the GEBV of animal *i* for crossbred performance and *w* ($w = 0, 0.25, 0.5, 0.75, {\text{or }}1$) is the weight that is assigned to GEBV for purebred and crossbred performance. With *w* = 0, both parental breeds were selected for purebred performance and with *w* = 1 selection within parental breeds was for crossbred performance.

### Genetic models

Three genetic models, which differed in the amount of dominance variance ($\sigma_{d}^{2}$) and in the proportion of QTL that showed over-dominance, were simulated to compare the performance of the above-mentioned selection criteria (Table 1). In Model 1, $\sigma_{d}^{2}$ of the trait of interest was equal to 0.1 and 25% of the QTL showed over-dominance. In Model 2, $\sigma_{d}^{2}$ was reduced to 0.05, and as a result, 10% of QTL showed over-dominance. Finally, in Model 3, $\sigma_{d}^{2}$ was equal to 0.1 as in Model 1, but no QTL showed over-dominance. For these three genetic models, training was on performance of purebred animals. However, to evaluate whether the ranking of selection criteria depends on the type of training population, we also simulated Model 1 with training on crossbred performance. Phenotypic variance ($\sigma_{p}^{2}$) was equal to 1 and narrow-sense heritability (*h*^2^) was equal to 0.3 in all cases.

**Table 1:** Genetic models used for simulation

Model	Training	Dominance variance	Overdominance (%)
1	Purebred	0.1	~25
1	Crossbred	0.1	~25
2	Purebred	0.05	~10
3	Purebred	0.1	0 (No overdominance)

### Population structure

A historical population of 2000 unrelated individuals was stochastically simulated and used as the ancestral population of two pure breeds (referred to as breeds A and B hereafter) that were used to create crossbreds. The historical population was randomly mated for 2000 generations. To simulate the two purebred populations, at generation 2000, two independent random samples of 100 animals were drawn from the last generation of the historical population, and each was randomly mated for another 100 generations. In subsequent generations, a two-way crossbreeding program with 40 generations of selection was simulated. From generation 1 to 40, 300 animals (the top 100 males and top 200 females) were selected from the 1000 available candidates in each parental breed, based on the selection criteria described above. The selected animals were randomly mated within each breed to produce 1000 purebred replacement animals for the next generation. Meanwhile, the 100 selected males of breed A were randomly mated to the 200 selected females of breed B to produce 1000 crossbred progeny. For all selection criteria and each model, breed A acted as the sire breed and breed B as the dam breed. The goal was to improve CP through selection in both parental breeds. The phenotypic mean of crossbreds was computed for each generation of selection (AB_1_ to AB_40_) to evaluate the realized cumulative response to selection. In models with training on purebred performance, A and B purebred datasets of 1000 animals each were used separately as training populations to estimate marker effects that were specific to that breed. In Model 1 with crossbred training, 2000 randomly selected AB crossbreds were used to estimate marker effects, which were then used to calculate genomic breeding values of animals in the parental breeds. In both types of training datasets (purebred and crossbred), training was repeated in each generation of selection, using the animals of the last generation only (more details about the population structure are in [4]).

### Genome and trait phenotypes

We considered a genome that comprised four chromosomes of 1 M each. This small genome size was chosen to limit computing time. In total, 400 segregating QTL and 4000 SNPs were simulated. Within a chromosome, the positions of 1000 SNPs and 100 QTL were randomly set. To obtain the required number of segregating loci after 2000 generations, twice as many bi-allelic loci were simulated by sampling initial allele frequencies from a uniform distribution and applying a recurrent mutation rate of 2.5 × 10^−5^. Mutation rates of loci were determined in preliminary analyses based on the number of polymorphic loci in generation 2000 necessary to obtain 4000 polymorphic SNPs and 400 QTL. SNPs and QTL were distinct loci and were randomly drawn from segregating loci, with a minor allele frequency (MAF) higher than 0.05 in generation 2000. The additive effect (*a*) of a QTL, defined as half the difference in genotypic value between alternate homozygotes, was sampled from a gamma distribution (0.4, 1.66). Dominance effects (*d*) were defined as the deviation of the genotypic value of the heterozygote from the mean of the genotypic values of the two homozygotes. Similar to Wellmann and Bennewitz [12, 13], first, the degree of dominance at the *i*th QTL (*h*_*i*_) was sampled from a normal distribution, $N\left( {0.5, 1} \right)$, and then dominance effects were calculated as $d_{i} = h_{i} .\left| {a_{i} } \right|$, where $\left| {a_{i} } \right|$ is the absolute value of the additive effect for each QTL. Thus, the absolute magnitudes of additive and dominance effects were not independent, i.e. loci with large additive effects were also more likely to have large dominance effects. To avoid QTL with over-dominance effects in Model 3, dominance effects for QTL that were sampled to have over-dominance effects were set equal to the absolute additive effect of the QTL.

Following simulation of additive and dominance QTL effects, additive and dominance variances were calculated and effects were scaled using an iterative procedure to reach the desired variances. In all models, additive and dominance effects of QTL alleles were assumed to be the same in the two breeds. Thus, G × E interactions and epistasis were not simulated. The phenotypes of the trait were simulated by adding a standard normal residual effect to the genotypic value of each animal.

### True and genomic estimated breeding values

Two types of true breeding values (TBV) were calculated, i.e. TBV for purebred performance (TBV-P) and TBV for crossbred performance (TBV-C). The TBV were calculated as the expected genotypic value of the offspring of a parent that carries a certain QTL-genotype, when this parent is mated at random to its own breed (TBV-P) or to the other pure breed (TBV-C). Thus, for animal *i* from breed *r*, the TBV for purebred performance was calculated as:

1 $${\text{TBVP}}_{ir} = \mathop \sum \limits_{j = 1}^{400} [(x_{ij} )(p_{jr} a_{j} + q_{jr} d_{j} )] + [(1 - x_{ij} )( - q_{jr} a_{j} + p_{jr} d_{j} )],$$

where *x*_*ij*_ is the proportion of alleles *A* that the individual carries (1 for *AA*, 0.5 for *Aa* and 0 for *aa*). Moreover, *p*_*jr*_ and *q*_*jr*_ are the allele frequencies (for *A* and *a*) for the *j*th QTL in breed *r*, and *a*_*j*_ and *d*_*j*_ are the true additive and dominance effects of the *j*th QTL. For example, for an *AA* parent at locus *j*, a fraction *p*_*jr*_ of its offspring will have genotype *AA*, while a fraction *q*_*jr*_ of its offspring will have genotype *Aa*. Hence, for locus *j*, the breeding value of this parent equals (*p*_*jr*_*a*_*j*_ + *q*_*jr*_*d*_*j*_), which is the first term in Eq. 1.

The expected genotype frequencies of crossbred offspring of a parent depend on the allele frequencies in the other pure breed (denoted $r^{{\prime }}$ here). Thus, for animal *i* from breed *r*, the TBV for CP was calculated using Eq. 1 but with *p*_*jr*_ and *q*_*jr*_ replaced by *p*_*jŕ*_ and *q*_*jŕ*_, where *p*_*jŕ*_ and *q*_*jŕ*_ are the allele frequencies (*A* and *a*) for the *j*th QTL in breed $r^{{\prime }}$.

Genomic estimated breeding values were calculated in the same way, but SNP genotypes were used rather than QTL genotypes, and estimated rather than true effects were used. Thus, from the estimates of additive ($\hat{a}$) and dominance effects ($\hat{d}$), the GEBV-P (for purebred performance) for animal *i* from breed *r* was calculated as:

2 $$\begin{aligned} & {\text{GEBV}}P_{ir} = \mathop \sum \limits_{j = 1}^{4000} [\left( {x_{ij} } \right)(p_{jr} \hat{a}_{j} + q_{jr} \hat{d}_{j} )] \\ & \quad + [\left( {1 - x_{ij} } \right)( - q_{jr} \hat{a}_{j} + p_{jr} \hat{d}_{j} )]. \\ \end{aligned}$$

For the calculation of GEBV-C (for crossbred performance), SNP frequencies in the other breed were used, i.e. *p*_*jr*_ and *q*_*jr*_ in Eq. 2 were replaced by *p*_*jŕ*_ and *q*_*jŕ*_ where *p*_*jŕ*_ and *q*_*jŕ*_ are the allele frequencies (*A* and *a*) for the *j*th SNP in breed $r^{{\prime }}$. SNP frequencies in the other breed were calculated based on SNP genotypes of all selection candidates in that breed.

### Estimation of marker effects

Bayesian ridge regression implemented in the BGLR “Bayesian general linear regression” R package was used to predict effects of SNPs [14]. The following model was used to predict the genetic effects associated with each SNP:

$$y_{i} = \mu + \sum X_{ij} a_{j} + \sum Z_{ij} d_{j} + e_{i} ,$$

where *y*_*i*_ is the phenotypic value of individual *i* in the training data, *μ* is the overall mean, *X*_*ij*_ is the copy number of a given allele of marker *j,* coded 0, 1 and 2 for *aa*, *aA* and *AA*, respectively, *a*_*j*_ is the random unknown additive effect for marker *j*, *Z*_*ij*_ is the indicator variable for heterozygosity of individual *i* at marker *j*, with *Z*_*ij*_ = *0* when individual *i* is homozygous at SNP *j* (*aa* or *AA*) and *Z*_*ij*_ = 1 if individual *i* is heterozygous at SNP *j* (*aA*), d_*j*_ is the random unknown dominance effect for SNP *j*, *e*_*i*_ is the residual effect for animal *i*, and Σ denotes summation over all SNPs *j*. For each analysis, the Gibbs sampler was run for 20,000 iterations, with the first 3000 discarded as burn in. Convergence of the resulting posterior distributions was assessed by the Heidelberger and Welch and the Geweke diagnostics using the Coda package [15].

### Analysis of correlation of LD phase

Correlation of LD phase between pure breeds A and B and their crossbred descendants was estimated to evaluate the degree of relatedness between the populations. To estimate this correlation, only segregating SNPs with a MAF higher than 0.01 in each population were included in the analysis. The correlation was estimated following Badke et al. [16] as:

$$R_{XY} = \frac{{\mathop \sum \nolimits_{{\left( {i,j} \right) \in p}} (r_{ij\left( X \right)} - \bar{r}_{X} )(r_{ij\left( Y \right)} - \bar{r}_{Y} )}}{sd\left( X \right)sd\left( Y \right)}$$

where *R*_*XY*_ is the correlation between $r_{{ij_{\left( X \right)} }}$ in population *X* and $r_{{ij_{\left( Y \right)} }}$ in population *Y*, $r_{{ij_{\left( Y \right)} }}$ is the correlation coefficient as a measure of LD between SNPs *i* and *j* in population *Y*, *sd*(*X*) and *sd*(*Y*) are the standard deviations of $r_{ij\left( X \right)}$ and $r_{ij\left( Y \right)}$, respectively, and $\bar{r}_{X}$ and $\bar{r}_{Y}$ are the average *r*_*ij*_ across all pairs of SNPs *i* and *j* within an interval of *p* for populations *X* and *Y*, respectively. Positive *R*_*XY*_ are expected when SNPs are in LD and show equal linkage phase in the two studied populations. Pairs of SNPs were binned according to distances between SNPs (intervals of 0.1 cM from 0 up to 10 cM) and average values of *R*_*XY*_ were calculated for each bin.

## Results

### Response to selection in crossbred performance

**Fig. 1:**
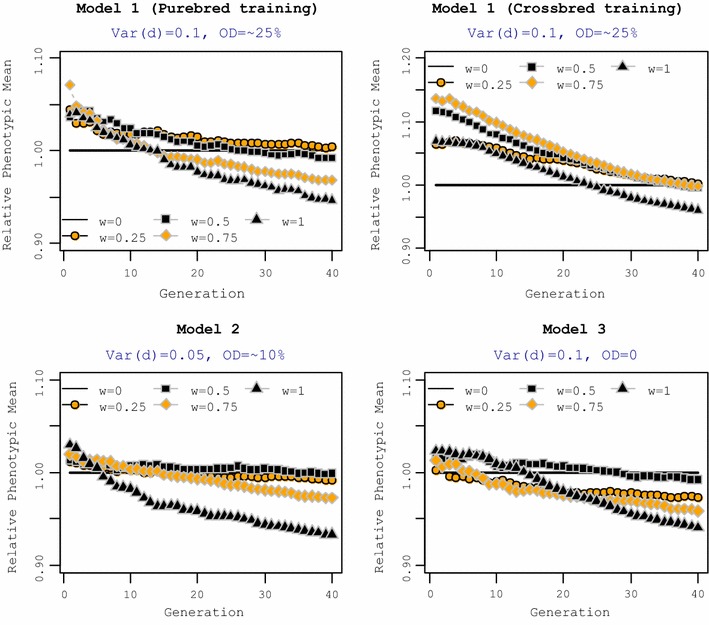
Mean phenotype of crossbred animals for different selection criteria relative to the reference selection criteria (*w* = 0). Mean phenotype of crossbreds for each selection criterion was plotted relative to the reference selection criterion (*w* = 0). The general criterion for selection of purebred parents was $SC_{i} = \left( {1 - w} \right) \cdot {\text{GEBV}}_{iP} + w \cdot {\text{GEBV}}_{iC}$. Training in Models 2 and 3 was on purebred animals. OD = % QTL with over-dominance

The phenotypic mean of crossbred animals was measured across 40 generations of selection for each of the five selection criteria and for each genetic model (Fig. 1). The mean phenotype of crossbreds for each selection criterion was expressed relative to the mean for the reference selection criterion (*w* = 0). For genetic Model 1 with training on purebred performance, all selection criteria with a non-zero *w* resulted in a greater response in CP in the short term than the reference selection criterion (*w* = 0). This superiority in CP was observed for at least 10 generations. However, the long-term response in CP differed between the selection criteria applied. Although the reference selection criterion (*w* = 0) resulted in the smallest CP in the short term, it realized a greater long-term response in CP than selection criteria with a weight on CP (*w* > 0.25). Setting *w* = 1, which means that animals were selected explicitly for CP, resulted in the smallest response in CP in the long term, whereas *w* = 0.25 resulted in the greatest response in the long term.

For genetic Model 1, with training on crossbred animals, shifting the selection criterion from purebred to crossbred performance led to a substantial improvement in CP, both in the short and long term. Compared to the reference selection criterion (*w* = 0), selection criteria with *w* ≥ 0.25 had a 6 to 13% greater response in CP in the short term, but this superiority decreased over generations. In the long term, the selection criterion with *w* = 1 resulted in a smaller response in CP than the reference selection criterion (*w* = 0). For genetic Model 2, which had lower dominance variance and, therefore, less over-dominance, selection criteria with a weight on CP resulted in a greater short-term response in CP than the reference selection criterion (*w* = 0) but only for a few initial generations. For example, *w* = 1 led to a response in CP that dropped below that of the reference selection criterion after five generations of selection. In the long term, selection criteria with high weights on CP (*w* ≥ 0.75) realized much less response in CP than the reference selection criterion. Selection criteria with *w* = 0.25 and 0.5 led to responses in CP that were comparable to response of the reference selection criterion in the long term.

Without over-dominance (genetic Model 3), in the short term, selection on CP (*w* = 1) led to a slightly greater response in CP than selection on the reference selection criterion (i.e. 2% more. In the short term, responses in CP were similar for *w* = 0.5 and 1. In the long term, similar to Model 1, the selection criterion with *w* = 1 on CP led to the smallest response in CP. Selection criteria with equal weights on purebred and crossbred performance (*w* = 0.5) resulted in a similar response in CP in the long term as the reference selection criterion, but greater CP in the short term.

**Fig. 2:**
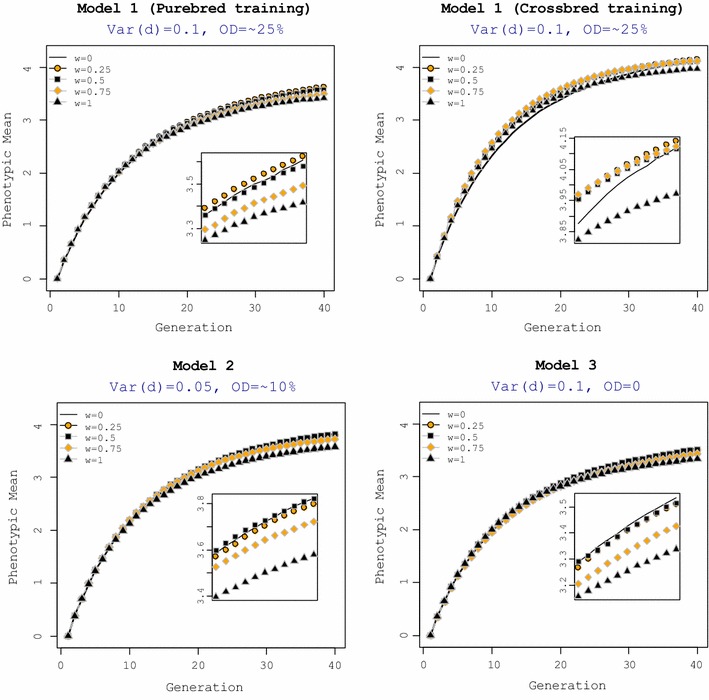
Cumulative response to selection. The general criterion for selection of purebred parents was $SC_{i} = \left( {1 - w} \right) \cdot {\text{GEBV}}_{iP} + w \cdot {\text{GEBV}}_{iC}$. Training under genetic Models 2 and 3 was on purebred animals. OD =  % QTL with over-dominance. Inset plots show the mean performance of crossbred animals under each model for generations 30 to 40

Figure 2 shows realized cumulative responses to selection in crossbred animals over 40 generations for the five selection criteria and each genetic model. For all models, selection criteria with a zero or small weight on CP realized higher response in CP in the long term, whereas explicit selection on crossbred performance, realized the smallest long-term response in CP.

In summary, for all genetic models, selection for purebred performance (reference selection criterion) resulted in a smaller response in CP in the short term but in the long term, this selection criterion was beneficial. In contrast, explicit selection of the pure breeds for CP (*w* = 1), although beneficial for CP in the short term, could not sustain this superiority in the long term.

### Purebred versus crossbred training population

**Fig. 3:**
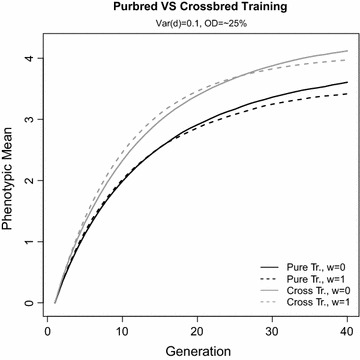
Mean phenotype of crossbred animals over generations under genetic Model 1. Two extreme values of *w* in the selection criteria (*w* = 0 and 1) were compared for purebred and crossbred training

Genetic Model 1 was used to compare the effect of the type of training population, i.e. using purebred and crossbred performance, on response in CP. Figure 3 shows the mean phenotype of crossbred animals over 40 generations for two extreme values of *w* for the selection criterion (*w* = 0 and 1). For both these selection criteria, training on crossbreds resulted in greater response to selection in CP than training on each purebred separately. Also, for both types of training, while selection for CP (*w* = 1) was beneficial in the short term, long-term response was greater with selection for purebred performance (*w* = 0).

### Response to selection in purebreds

**Fig. 4:**
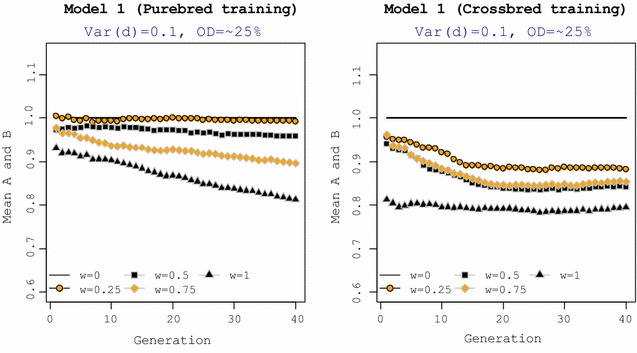
Mean phenotypic average of pure breeds. Mean phenotype of pure breeds are expressed relative to the means obtained with the reference selection criterion (*w* = 0). The general criterion for selection of purebred parents was $SC_{i} = \left( {1 - w} \right) \cdot {\text{GEBV}}_{iP} + w \cdot {\text{GEBV}}_{iC}$. Training under genetic Models 2 and 3 was on purebred data. OD = % QTL with over-dominance

Response to selection averaged across breeds A and B for 40 generations of selection under genetic Model 1 is presented in Fig. 4. The mean phenotype of purebred animals for different selection criteria is plotted relative to mean phenotype for the reference selection criterion (*w* = 0). Since results with Models 2 and 3 were similar to those with Model 1 with purebred training, they are not shown. For all genetic models, the reference selection criterion with *w* = 0 for CP resulted in the greatest response, both in the short and long term, whereas *w* = 1 resulted in the smallest response. In Model 1 with purebred training, putting a relatively small weight on CP (*w* = 0.25) did not affect response in purebreds and the mean performance of purebreds for this selection criterion was comparable with that for the reference selection criterion. Training on crossbreds under Model 1 resulted in lower performance of purebreds compared to the reference selection criterion when *w* > 0. In general, for all genetic models, shifting from selection on purebred performance to crossbred performance, i.e. increasing *w* from 0 to 1, reduced response in purebred performance.

### Heterosis in crossbreds

**Fig. 5:**
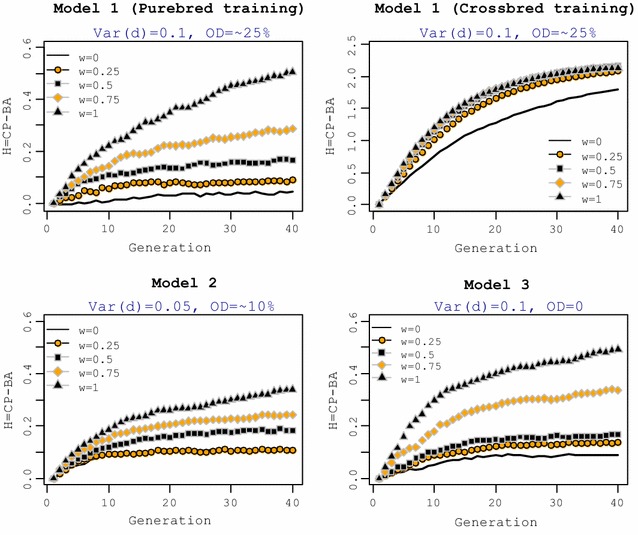
Heterosis in crossbreds. The amount of heterosis obtained with each selection criterion was calculated as the difference between crossbred performance (CP) and breed average (BA) over generations. The general criterion for selection of purebred parents was $SC_{i} = \left( {1 - w} \right) \cdot {\text{GEBV}}_{iP} + w \cdot {\text{GEBV}}_{iC}$. Training under genetic Models 2 and 3 was on purebred data. OD = % QTL with over-dominance

Heterosis refers to the superior performance of crossbred animals compared to the average performance of their purebred parents. The amount of heterosis achieved for each selection criterion was calculated as the difference between CP and BA over generations (H = CP–BA) (Fig. 5).

For all genetic models, the selection criterion with *w* = 1 exploited more heterosis in crossbreds than other selection criteria and the amount of heterosis increased over generations with this selection criterion. In contrast, the reference selection criterion with selection for purebred performance (*w* = 0) resulted in the smallest amount of heterosis over generations for all genetic models. Compared to genetic Model 1 with purebred training, reducing dominance variance (Model 2) and absence of over-dominance (Model 3) did not affect the ranking of selection criteria in terms of heterosis. For all genetic models, shifting from selecting on purebred to crossbred performance (i.e. from *w* = 0 to 1) increased the amount of heterosis observed in crossbreds. For genetic Model 1, with crossbred training, heterosis increased over generations for all selection criteria. The difference between selection criteria with *w* > 0 was negligible and the reference selection criterion, i.e. selecting on purebred performance also increased heterosis in crossbreds.

### Fixation of alleles

Table 2 summarizes the percentages of QTL alleles that became fixed for the five selection criteria and all genetic models. Total fixation was calculated as the percentage of QTL that was fixed for either allele in the last generation, averaged over the two breeds. Increasing the weight (*w*) in the selection criterion increased the percentage of allele fixation. In all models, the selection criterion with *w* = 1 had the highest percentage of QTL allele fixation, while the reference selection criterion with selection on purebred performance had the lowest allele fixation.

**Table 2:** Average percentage of QTL fixation in generation 40 across the two parental breeds for different selection criteria and genetic models

Model		*w* = 0	*w* = 0.25	*w* = 0.5	*w* = 0.75	*w* = 1
Model 1 (Purebred training)	Total	33.37	35.25	36.12	38.25	40.37
	Common	70.14	68.29	67.48	65.23	62.80
	Alternate	29.85	31.70	32.51	34.76	37.19
	Favorable	63.77	63.63	63.07	62.61	61.09
	Unfavorable	36.22	36.36	39.92	37.40	38.91
Model 1 (Crossbred training)	Total	41.50	46.12	49.00	50.00	50.50
	Common	61.28	57.30	56.58	55.93	55.48
	Alternate	38.71	42.69	43.41	44.06	44.51
	Favorable	60.39	59.82	59.46	59.00	58.55
	Unfavorable	39.60	40.17	40.53	40.92	41.44
Model 2	Total	39.00	40.00	40.87	42.37	42.62
	Common	67.23	66.64	65.63	64.23	62.16
	Alternate	32.76	33.35	34.36	35.77	37.83
	Favorable	63.25	63.27	63.13	61.75	61.20
	Unfavorable	36.74	36.72	36.86	38.24	38.81
Model 3	Total	31.00	32.87	34.75	36.50	38.87
	Common	72.47	70.69	69.15	66.26	63.46
	Alternate	27.52	29.30	30.84	33.73	36.53
	Favorable	65.03	64.75	64.31	63.24	62.17
	Unfavorable	34.96	35.25	35.69	36.75	37.83

Total fixation was calculated as the percentage of QTL that were fixed for either allele in the last generation. The general criterion for the selection of purebred parents was $SC_{i} = \left( {1 - w} \right) \cdot {\text{GEBV}}_{iP} + w \cdot {\text{GEBV}}_{iC}$. Training in genetic Models 2 and 3 was on purebred data

The percentage of common (alternate) allele fixation represents the number of QTL that were fixed for the same (alternate) allele in the two breeds in the last generation. Across all genetic models, selection on purebred performance in each pure line (*w* = 0) resulted in more frequent fixation of the same allele in both pure breeds, while shifting the selection criteria from purebred to crossbred selection (i.e. increasing *w*) reduced the probability of fixation of the same allele in the two pure breeds. In contrast, with full weight on crossbred performance (*w* = 1), the two breeds were more often fixed for alternate alleles.

The selection criteria also differed in the percentage of QTL that were fixed for the favorable allele, which was defined based on the sign of the additive effect of the allele. Increasing the weight on CP resulted in slightly less fixation of favorable alleles in the pure breeds. However, fixation of unfavorable alleles (i.e. loss of favorable alleles) in pure breeds increased by shifting selection from purebred to crossbred performance. In addition, training on CP increased fixation of unfavorable alleles compared to training on purebred performance.

### Over-dominance fixation

**Fig. 6:**
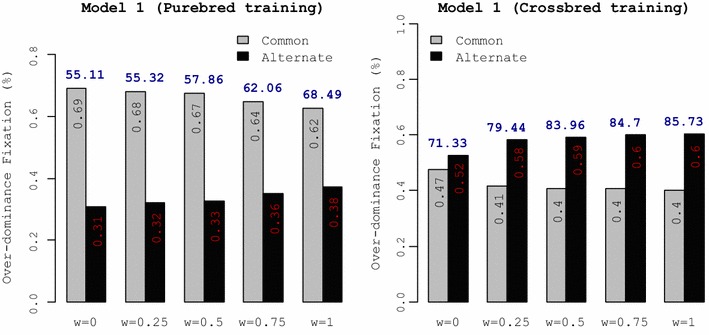
Fixation of over-dominant QTL in the parental breeds. The percentages (inside bars) indicate the proportion of fixed over-dominant QTL that are fixed for the same or alternate alleles in the parental breeds. Bolded blue values above bars indicate the total fixation of over-dominant QTL in generation 40 for each selection criteria. The general criterions for selection of purebred parents was $SC_{i} = \left( {1 - w} \right) \cdot {\text{GEBV}}_{iP} + w \cdot {\text{GEBV}}_{iC}$

Percentages of fixation of over-dominant QTL in the two pure breeds in the last generation are in Fig. 6. For both types of training, selection on CP (*w* = 1) resulted in a greater percentage of fixation of over-dominant QTL (68.5 and 85.7% for purebred and crossbred training, respectively), while selection for purebred performance (*w* = 0) resulted in less fixation of over-dominant QTL (55.1 and 71.3% for purebred and crossbred training, respectively). Total fixation for both types of training increased by shifting selection from purebred to crossbred performance. In addition, compared to purebred training, crossbred training generally led to more fixation of over-dominant QTL.

The percentages of over-dominant QTL that were fixed for either the same or alternate alleles in the two pure breeds are also presented in Fig. 6. For genetic Model 1 with purebred training, changing the selection criteria from purebred to crossbred performance decreased the percentage of fixation of common alleles for over-dominant QTL and increased the percentage of fixation of alternate alleles in the pure breeds, with selection for CP (*w* = 1) resulting in the highest percentage of fixation of alternate alleles.

Fixation of common and alternate alleles of over-dominant QTL under genetic Model 1 was also associated with the type of training; with crossbred training, the percentage of fixation was higher for alternate alleles than for common alleles for all selection criteria. In addition, changing from selection on purebred to crossbred performance resulted in a higher (lower) percentage of fixation of alternate (common) alleles in the pure breeds.

### Accuracy of selection

**Fig. 7:**
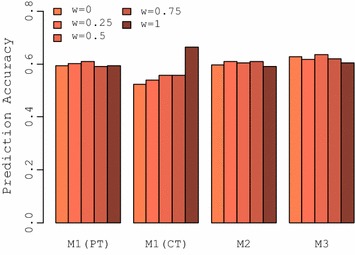
Accuracy of selection for five selection criteria for each genetic model. Accuracies are presented for breed A only. **M1 (PT)**: genetic Model 1 with purebred training, **M1 (CT)**: genetic Model 1 with crossbred training

Prediction accuracy, i.e. correlation between the breeding values predicted by genomic selection and the true breeding value obtained from simulation, ranged from 0.52 to 0.66 in the first generation across the genetic models (Fig. 7). Note that accuracies in Fig. 7 refer to the correlation between the selection criterion and TBV-C. In other words, when in the selection criterion *w* is set to 0, selection is on purebred performance but the accuracy is the correlation of GEBV-P with TBV-C, while accuracy is the correlation between GEBV-C and TBV-C when selection is on CP (*w* = 1). Generally, accuracies with purebred training were not affected by the value of *w* in the selection criterion. For genetic Model 1 with crossbred training, selection criteria with *w* = 0 and 1 had the lowest and highest accuracy, respectively. Thus, predicting GEBV-P based on crossbred performance is more difficult than predicting GEBV-P on purebred performance. Similarly, predicting GEBV-C is more effective when based on crossbred performance than based on purebred performance.

### Correlation of LD phase

**Fig. 8:**
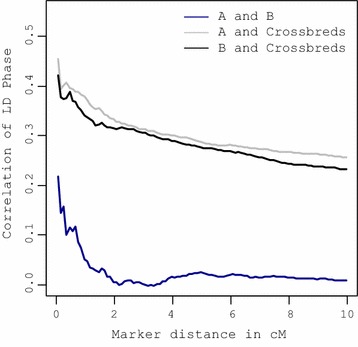
Correlation of LD phase (*R*_*XY*_) between populations for SNP pairs for different distances between SNPs

The correlation of LD phase between breeds A and B was low and decreased rapidly with increasing distance between SNPs (Fig. 8). For SNPs less than 0.5 cM apart, the mean correlation was 0.15 and decreased towards 0 at distances of 10 cM. In contrast, the correlation of LD phase between purebred populations and their crossbred descendants was high; the correlation was equal to 0.46 between breed A and the crossbred population for SNPs less than 0.5 cM and decreased to 0.25 for SNPs 10 cM apart. Corresponding correlations for breed B were 0.43 and 0.23 for SNPs 0.5 and 10 cM apart, respectively.

## Discussion

We investigated the performance of crossbred progeny in a two-way crossbreeding program over 40 generations. Purebred parents in each generation were selected based on criteria that ranged from a selection for purebred performance to selection for crossbred performance, using different weights on each. We showed that selection in pure breeds using a selection criterion that specifically targets CP, in spite of being beneficial in the short term, results in smaller performance of crossbred animals in the long term. These results indicate that a selection criterion that properly weights purebred and crossbred performance can be used to maximize response over a given time horizon.

### Comparison of selection criteria

We investigated five selection criteria that differed in the weights on and ability to exploit the two components that determine the merit of crossbreds, i.e., parental breed average and heterosis. When selecting on purebred performance (reference selection criterion), selection was on breed average and, led to the highest mean performance of purebred breeds over generations. However, since this selection criterion (*w* = 0) resulted in less progress in heterosis, the overall performance of crossbreds was lower in the short to medium term. The inability of the reference selection criterion to increase heterosis significantly can be explained as follows. In our study, we assumed that the additive and dominance effects of QTL were the same in both breeds. With selection for purebred performance, the same alleles were often fixed in the two parental breeds. For all genetic models considered, the highest proportion of QTL fixed for the same allele in the two breeds relative to the total number of fixed QTL in the last generation was obtained with the reference selection criterion (Table 2). Fixation of the same alleles in the two breeds reduces the heterozygosity in crossbreds and heterosis. However, in the long term, this selection criterion realized the largest response in crossbreds because a higher percentage of favorable alleles was fixed within the pure breeds, resulting in more genetic gain in pure breeds and higher performance of crossbred progeny.

When *w* increased from 0 to 1 in the selection criterion, selection changed from purebred to crossbred selection by putting more weight on ${\text{GEBV}}_{iC}$. As a result, heterosis was exploited more effectively by selection criteria, which increased performance of crossbreds in the short to medium term compared to the reference selection criterion. Since heterosis depends on differences in allele frequencies between the parental breeds, these results suggest that selection based on ${\text{GEBV}}_{iC }$ drives allele frequencies in opposite directions in the two parental breeds or at least causes divergence in allele frequencies between the parental breeds. As a result, selection based on ${\text{GEBV}}_{iC }$ led to more fixation of alternate QTL alleles in the two parental breeds, which is beneficial for heterosis in crossbreds in the presence of dominance. In addition, compared to the reference selection criterion, for over-dominant QTL the parental breeds were more often fixed for alternate alleles when ${\text{GEBV}}_{iC }$ was included in the selection criterion (Fig. 6), which explains the greater heterosis obtained with these selection criteria. However, in the long term, selection criteria with non-zero *w* resulted in smaller CP. Although changing the selection criteria from selection on purebred performance to crossbred performance allowed the non-additive genetic effects (heterosis) to be exploited in crossbred merit more effectively, it also resulted in lower genetic gain in the pure breeds. In fact, regardless of whether selection is on ${\text{GEBV}}_{iC }$ or ${\text{GEBV}}_{iP}$, both components of crossbred merit (${\text{CP}} = {\text{BA}} + {\text{H}})$ are involved in determining the merit of crossbreds. However, the potential of ${\text{GEBV}}_{iC }$ and ${\text{GEBV}}_{iP}$, to exploit breed average versus heterosis differs. While ${\text{GEBV}}_{iP} ,$ is more capable of improving the additive component (BA), ${\text{GEBV}}_{iC }$ exploits the non-additive component (H) more effectively. Comparing short and long-term responses shows that, although heterosis is beneficial for crossbred performance in the short term, the main component that determines long-term performance of crossbreds is BA rather than H.

Including CP in the selection criteria resulted in greater short- to medium-term response in crossbred animals compared to selection on purebred performance only. Consideration of cumulated discounted response favors schemes that lead to a greater response in early generations, in particular if the discount rates is high. Thus, for commercial competitive breeding, our results indicate an advantage of including CP in the selection criterion. In practice, a breeder is interested in genetic improvement of both purebred and crossbred performance and, thus, in a breeding objective that focuses both on purebred and crossbred performance. One approach to address that objective would be to use a selection criterion with *w* = 0.25 or 0.5, as in this study, which puts weight on both purebred and crossbred breeding values of the purebred parents and, thus, in simultaneous improvement of purebred and crossbred performance. Compared to the reference selection criterion, these selection criteria result in greater short-term performance in CP and nearly identical or comparable long-term performance. In addition, purebred performance was only slightly (2 to 3%) smaller than obtained with the reference selection across generations. Thus, by putting appropriate weights on ${\text{GEBV}}_{C}$ and ${\text{GEBV}}_{P}$ in the selection criterion, simultaneous progress in purebreds and crossbreds may be guaranteed, with additional short-term gain being achieved with no loss in long-term gain.

### Comparison of genetic models

In Model 1, dominance variance was one-third as large as the additive genetic variance, which is within the range reported for many traits in livestock [17–20] and plant breeding populations [21–23]. However, because of the wide range of reported proportions of dominance variance across traits and populations, dominance variance was reduced to 5% in Model 2 to investigate if the amount of dominance variance affects the performance of different selection criteria. As for Model 1, for Model 2, selection criteria with a weight on ${\text{GEBV}}_{C}$ (*w* ≥ 0.25) improved short-term performance in CP more than the reference selection criterion but to a lesser degree than Model 1 and performance fell below that of the reference selection criterion in fewer generations. For example, the selection criterion with *w* = 1 with purebred training resulted in greater response in CP than the reference selection criterion for 13 generations for Model 1 but only for five generations for Model 2. Since the lower dominance variance for Model 2 compared to Model 1 also results in a lower percentage of over-dominant QTL, we hypothesized that over-dominance may contribute to the effect of including CP in the selection criterion on response in CP.

Over-dominant QTL have been identified in livestock and plants for economically important traits [24–28]. Although the percentage of QTL showing over-dominance has not been clearly determined for complex traits in livestock, they have been shown to be frequent in plants, especially for reproductive traits. For example, Lu et al. [29] studied four traits in two back-cross populations of maize and, based on the absolute value of *d*/*a*, 24 of the 28 QTL (86%) identified for grain yield showed over-dominance. For three non-reproductive traits, fewer QTL with over-dominance were identified, i.e. two out of 16 (12.5%) for grain moisture, one out of 8 (12.5%) for stalk lodging, and four out of 11 (36%) for plant height. The association of over-dominance with reproductive traits has also been reported for laboratory animals. A QTL-mapping study in an F2 cross between two mouse strains measured 17 body composition and growth traits and identified 139 QTL [30], of which 9% showed over-dominance ($\frac{d}{a} > 1$ or $\frac{d}{a} < - 1$). Another study on five reproductive traits found that seven of the 15 QTL detected showed over-dominance [31].

Regardless of their frequency of occurrence, QTL that exhibit over-dominance have a relatively large effect on the amount of heterosis exhibited by a trait. Thus, in Model 3, in order to investigate the potential effect of over-dominance on performance of the selection criteria, we excluded over-dominant QTL in the genetic architecture of the trait but without changing the dominance variance. However, even without over-dominance, selection criteria that included CP realized greater response in CP, although their relative superiority was lower than for Model 1. In addition, a selection criterion with a relatively small weight on CP (*w* = 0.25), did not improve response in CP compared to the reference selection criterion. In conclusion, our results of the comparison between Models 3 and 1, with absence of over-dominance being the only difference, suggest that over-dominance contributes to the greater response in CP observed for selection criteria that include CP.

Comparison of Model 1 with training on purebred versus crossbred data showed that selection criteria with *w* ≥ 0.25 resulted in greater response in CP than the reference selection criterion. With purebred training, these selection criteria were about 5% superior to the reference selection criterion in the short term, but this superiority increased to 14% and remained for more generations with training on crossbreds. This higher superiority appears to be due to the larger amount of heterosis obtained with crossbred training. For Model 1, regardless of the selection criterion, the percentage of QTL that were fixed for the alternate allele in the two parental breeds was higher with crossbred training than with purebred training, which explains the larger amount of heterosis that was observed in crossbreds. In addition, in contrast to purebred training, with crossbred training, more over-dominant QTL were fixed for alternate alleles than for the same allele in the parental breeds (Fig. 6), which explains the greater heterosis observed in crossbreds with crossbred training. In addition, in order to investigate the potential effect of over-dominant QTL on the observed superiority of selection criteria that included CP, over-dominance was not allowed in the genetic structure of the trait, as in Model 3 (see Additional file 1: Figure S1). Results showed that absence of over-dominance did not affect the performance of selection criteria with *w* ≥ 0.25 and these selection criteria were superior to the reference selection criterion not only in the short and medium term but also in the long term (except for *w* = 1). It should be noted that, although over-dominance was absent in this model, QTL with complete and partial dominance were present and may contribute to the larger amount of heterosis observed in crossbreds in these cases.

### Purebred versus crossbred training population

Training on crossbred data for genomic selection of purebreds for CP has been suggested [1, 4]. It is expected that training on crossbred data accounts for the factors that cause the genetic correlation between purebreds and crossbreds (*r*_*pc*_) to be less than 1, which include non-additive effects (mainly dominance), genotype by environment interactions (G × E), breed of origin effects, and differences in allele frequencies between breeds. In this study, G × E interaction was not included in the simulations and, thus, the deviation of *r*_*pc*_ from 1 (0.82 ± 0.05 on average across Models) was purely the result of dominance effects and differences in allele frequencies between the two pure breeds. Our results showed that training on crossbreds resulted in a greater response to selection in CP than training on each purebred separately (Fig. 3). Previous simulation studies have shown that training on crossbred data by either ignoring [2, 3] or accounting [2, 4] for the breed origin of the alleles in crossbreds can be beneficial in crossbreeding programs. In fact, training on crossbreds by using an appropriate model, can account for most of the factors that cause *r*_*pc*_ to be lower than 1. Using real data, Xiang et al. [32] applied single-step best linear unbiased prediction (BLUP) to data on total number of piglets born in Danish Landrace, Yorkshire and two-way crossbred pigs. Their results confirmed that including crossbred genomic information improved reliabilities of genomic predictions of CP for purebred boars. Similarly, Iversen et al. [33] found that including the genotypes of crossbred animals in the genomic relationship matrix increased prediction accuracy of total number born and live born for both purebreds and crossbreds. Lopes et al. [34] showed that predicting performance of crossbred sows for litter size and gestation length was more accurate when training was performed on crossbred than on purebred data. In addition, they found evidence of breed-specific SNP effects by training on crossbred data, although prediction accuracies did not improve for the analyzed traits when this was accounted for. In summary, the results from both simulation studies and real data analyses indicate that compared to training on purebred data, training on crossbred data is beneficial in crossbreeding programs.

## Conclusions

Genomic selection of pure breeds with a selection criterion that specifically targets CP, although it is beneficial in the short to medium term, is inferior to purebred selection in the long term. A selection criterion that maximizes a combination of short- and longer-term responses for CP, must improve the components that define crossbred merit simultaneously, i.e., breed average and heterosis. To increase response to selection for CP, training on crossbred data is more effective than training on purebred data.

## Additional file

**Additional file 1. **Mean crossbred performance, mean phenotype of pure breeds and heterosis in crossbreds for Model 3 ($\sigma_{d}^{2}=0.1$ and absence of overdominance QTL) with crossbred training.

## References

[1] Dekkers JCM (2007). Marker-assisted selection for commercial crossbred performance. J Anim Sci.

[2] Ibánẽz-Escriche N, Fernando RL, Toosi A, Dekkers JC (2009). Genomic selection of purebreds for crossbred performance. Genet Sel Evol..

[3] Zeng J, Toosi A, Fernando RL, Dekkers JC, Garrick DJ (2013). Genomic selection of purebred animals for crossbred performance in the presence of dominant gene action. Genet Sel Evol..

[4] Esfandyari H, Sørensen AC, Bijma P (2015). A crossbred reference population can improve the response to genomic selection for crossbred performance. Genet Sel Evol..

[5] Dekkers JC, Chakraborty R (2004). Optimizing purebred selection for crossbred performance using QTL with different degrees of dominance. Genet Sel Evol..

[6] Falconer DS, Mackay TFC (2004). Introduction to quantitative genetics.

[7] Esfandyari H, Sørensen AC, Bijma P (2015). Maximizing crossbred performance through purebred genomic selection. Genet Sel Evol..

[8] Pong-Wong R, Woolliams JA (1998). Response to mass selection when an identified major gene is segregating. Genet Sel Evol..

[9] Larzul C, Manfredi E, Elsen JM (1997). Potential gain from including major gene information in breeding value estimation. Genet Sel Evol..

[10] Gibson J. Short-term gain at the expense of long-term response with selection of identified loci. In: Proceedings of the 5th world congress on genetics applied to livestock production: 7–12 August 1994; Guelph; 1994. Accessed 24 May 2017.

[11] Esfandyari H, Sørensen AC. Genomic simulation of purebred and crossbred populations [R package xbreed version 1.0.1]. Comprehensive R Archive Network (CRAN); 2017. Available from: https://cran.r-project.org/web/packages/xbreed/index.html.

[12] Wellmann R, Bennewitz J (2012). Bayesian models with dominance effects for genomic evaluation of quantitative traits. Genet Res (Camb)..

[13] Wellmann R, Bennewitz J (2011). The contribution of dominance to the understanding of quantitative genetic variation. Genet Res (Camb)..

[14] Perez P, de los Campo G (2014). Genome-wide regression and prediction with the BGLR statistical package. Genetics.

[15] Plummer M, Best N, Cowles K, Vines K (2006). CODA: convergence diagnosis and output analysis for MCMC. R News..

[16] Badke YM, Bates RO, Ernst CW, Schwab C, Steibel JP (2012). Estimation of linkage disequilibrium in four US pig breeds. BMC Genomics.

[17] Lopes MS, Bastiaansen JWM, Janss L, Knol EF, Bovenhuis H (2015). Estimation of additive, dominance, and imprinting genetic variance using genomic data. G3 (Bethesda).

[18] Ertl J, Legarra A, Vitezica ZG, Varona L, Edel C, Emmerling R (2014). Genomic analysis of dominance effects on milk production and conformation traits in Fleckvieh cattle. Genet Sel Evol..

[19] Sun C, VanRaden PM, Cole JB, O’Connell JR (2014). Improvement of prediction ability for genomic selection of dairy cattle by including dominance effects. PLoS One.

[20] Su G, Christensen OF, Ostersen T, Henryon M, Lund MS (2012). Estimating additive and non-additive genetic variances and predicting genetic merits using genome-wide dense single nucleotide polymorphism markers. PLoS One.

[21] Du FX, Hoeschele I (2000). Estimation of additive, dominance and epistatic variance components using finite locus models implemented with a single-site Gibbs and a descent graph sampler. Genet Res.

[22] Silva AR, Souza CL, Aguiar AM, De Souza AP (2004). Estimates of genetic variance and level of dominance in a tropical maize population. I. Grain yield and plant traits. Maydica..

[23] Wolf DP, Peternelli LA, Hallauer AR (2000). Estimates of genetic variance in an F2 maize population. J Hered.

[24] Shang L, Wang Y, Cai S, Wang X, Li Y, Abduweli A (2015). Partial dominance, overdominance, epistasis and QTL by environment interactions contribute to heterosis in two upland cotton hybrids. G3 (Bethesda).

[25] Li L, Lu K, Chen Z, Mu T, Hu Z, Li X (2008). Dominance, overdominance and epistasis condition the heterosis in two heterotic rice hybrids. Genetics.

[26] Komatsu M, Sato Y, Negami T, Terada T, Sasaki O, Yasuda J (2014). Overdominance effect of the bovine ghrelin receptor (*GHSR1a*)- *DelR242* locus on growth in Japanese shorthorn weaner bulls: heterozygote advantage in bull selection and molecular mechanisms. G3 (Bethesda).

[27] Gemmell NJ, Slate J (2006). Heterozygote advantage for fecundity. PLoS One.

[28] Kim KS, Kim JJ, Dekkers JM, Rothschild M (2004). Polar overdominant inheritance of a DLK1 polymorphism is associated with growth and fatness in pigs. Mamm Genome.

[29] Lu H, Romero-Severson J, Bernardo R (2003). Genetic basis of heterosis explored by simple sequence repeat markers in a random-mated maize population. Theor Appl Genet.

[30] Rocha JL, Eisen EJ, Van Vleck LD, Pomp D (2004). A large-sample QTL study in mice: I. Growth. Mamm Genome.

[31] Rocha JL, Eisen EJ, Siewerdt F, Van Vleck LD, Pomp D (2004). A large-sample QTL study in mice: III. Reproduction. Mamm Genome.

[32] Xiang T, Nielsen B, Su G, Legarra A, Christensen OF (2016). Application of single-step genomic evaluation for crossbred performance in pig. J Anim Sci.

[33] Iversen MW, Nordbø Ø, Gjerlaug-Enger E, Grindflek E, Lopes MS, Meuwissen THE (2017). Including crossbred pigs in the genomic relationship matrix through utilization of both linkage disequilibrium and linkage analysis. J Anim Sci.

[34] Lopes MS, Bovenhuis H, Hidalgo AM, van Arendonk JAM, Knol EF, Bastiaansen JWM (2017). Genomic selection for crossbred performance accounting for breed-specific effects. Genet Sel Evol..

