# Supplementary material for: Balanced selection on purebred and crossbred performance increases gain in crossbreds
Source: Genet Sel Evol. 2018 Mar 22;50:8. doi: 10.1186/s12711-018-0379-9 (PMC5865367; doi:10.1186/s12711-018-0379-9)
Supplement: Supplementary file 1 — Additional file 1. Mean crossbred performance, mean phenotype of pure breeds and heterosis in crossbreds for Model 3 ($\sigma_{d}^{2}=0.1$σd2=0.1 and absence of overdominance QTL) with crossbred training. [file 12711_2018_379_MOESM1_ESM.docx]

**Additional file 1**

**Mean crossbred performance, mean phenotype of pure breeds and heterosis in crossbreds for Model 3 (**$\sigma_{d}^{2}$**= 0.1 and absence of overdominance QTL) with crossbred training.**

|  |
| --- |
| Supplementary Fig. S1. Mean crossbred performance, mean phenotype of purebreds and heterosis in crossbreds for Model 3 ($\sigma_{d}^{2}$= 0.1 and absence of overdominance QTL) with crossbred training. The general criterion for selection of purebred parents was ${SC}_{i}={\left( 1-w \right).GEBV}_{iP}+w.{GEBV}_{iC}$. |
